# Local atomic order and hierarchical polar nanoregions in a classical relaxor ferroelectric

**DOI:** 10.1038/s41467-019-10665-4

**Published:** 2019-06-21

**Authors:** M. Eremenko, V. Krayzman, A. Bosak, H. Y. Playford, K. W. Chapman, J. C. Woicik, B. Ravel, I. Levin

**Affiliations:** 1 Materials Measurement Science Division, National Institute of Standards and Technology, Gaithersburg, MD USA; 2 European Synchrotron Radiation Facility, BP 22-, 38043 Grenoble, Cedex France; 3 ISIS Facility STFC ISIS Facility, Rutherford Appleton Laboratory, Oxfordshire, OX11 0QX UK; 4 Department of Chemistry, Stony Brook University, Stony Brook, NY USA

**Keywords:** Ferroelectrics and multiferroics, Characterization and analytical techniques

## Abstract

The development of useful structure-function relationships for materials that exhibit correlated nanoscale disorder requires adequately large atomistic models which today are obtained mainly via theoretical simulations. Here, we exploit our recent advances in structure-refinement methodology to demonstrate how such models can be derived directly from simultaneous fitting of 3D diffuse- and total-scattering data, and we use this approach to elucidate the complex nanoscale atomic correlations in the classical relaxor ferroelectric PbMg_1/3_Nb_2/3_O_3_ (PMN). Our results uncover details of ordering of Mg and Nb and reveal a hierarchical structure of polar nanoregions associated with the Pb and Nb displacements. The magnitudes of these displacements and their alignment vary smoothly across the nanoregion boundaries. No spatial correlations were found between the chemical ordering and the polar nanoregions. This work highlights a broadly applicable nanoscale structure-refinement method and provides insights into the structure of PMN that require rethinking its existing contentious models.

## Abstract (web summary)

The understanding of relaxor ferroelectrics is hindered by the complexity of nanoscale perturbations of their structure. Here, a data set of independent techniques treated on common footing provides a multiscale description of atomic order which reconciles conflicting models derived from single methods.

## Introduction

Atomic arrangements in most practical materials exhibit some degree of disorder. Glasses or amorphous matter lack structural coherence beyond the first few nearest neighbors, whereas in crystals the disorder exists as a perturbation of an otherwise periodic atomic array. Because of disorder, the local structure—a term that refers to atomic arrangements on a scale ranging from sub-nanometer to several nanometers—differs from the average. Evidence grows that such fine details of crystal structures, which are largely overlooked by traditional crystallographic methods, control the functional responses of many advanced technological materials. Therefore, the ability to consistently and accurately characterize local structure on the relevant length scales becomes critical for the informed design of materials for applications^1,2^.

Relaxor ferroelectrics^3–5^ are a classical example of systems with properties controlled by nanoscale atomic order that has remained ill-understood despite over a half century of intense studies using the most advanced measurement and computational techniques. Lead magnesium niobate, PbMg_1/3_Nb_2/3_O_3_ (PMN), and its solid solutions, which exhibit highly attractive piezoelectric characteristics, have been and remain at the center of the quest to understand the structural origins of the relaxor behavior. PMN crystallizes with a perovskite-like structure featuring a nanoscale ordering of Mg and Nb. The polarizable Nb and Pb cations are offset from their respective centrosymmetric positions, thus yielding local electric dipoles. While on average the PMN structure exhibits cubic symmetry (retained down to at least 5 K), such polar cation displacements are correlated over a local range; however, the exact nature of these correlations and their relations to the underlying short-range chemical order remain contentious.

Most commonly, the displacement correlations in PMN are perceived to be manifested as polar nanoregions (PNR), several nanometers in size, which emerge below the so-called Burns temperature of ≈620 K^6–8^ and are widely regarded as a key feature associated with the relaxor behavior. A formal definition of PNRs is missing but typically this term refers to finite-size regions, dynamic or static, having nonzero spontaneous electrical polarization. A large fraction of structural research on PMN has involved X-ray and neutron-scattering measurements on single crystals, with a focus on the interpretation of the rich diffuse-scattering patterns in terms of displacement correlations^8–18^; this diffuse scattering appears below the Burns temperature and becomes increasingly pronounced as temperature is reduced. Various models of PNRs that could give rise to these patterns have been proposed, ranging from static nanodomains having well-defined shapes and boundaries^18–20^ to dynamic displacement patterns controlled by acoustic^15^ and/or optical phonon modes^22^. Some works suggested that the PNRs are distributed in a disordered paraelectric matrix^19–21,23^, whereas recent molecular-dynamics studies^17^ have rejected this interpretation, claiming instead that PNRs are separated by domain walls. Similarly, while some computational studies associate the PNRs with chemically ordered regions (COR)^23^, others question this relation^15,22^. The debate continues in part because no comprehensive structural model of PMN that would provide at least a snapshot of the multiscale correlated disorder, while also accounting for all the available experimental data, has been reported thus far.

Here, we use PMN to demonstrate an approach for obtaining fully atomistic nanoscale structural models directly from the experimental data. We employ large atomic ensembles and a Reverse Monte Carlo (RMC) algorithm to simultaneously fit multiple types of diffraction and spectroscopic data, which notably include three-dimensional (3D) X-ray diffuse-scattering intensity distributions. Our results confirmed the rocksalt-type random-site chemical ordering of Mg and Nb and revealed a continuous distribution of the order parameter, with its nanoscale spatial fluctuations yielding regions of stronger order. The displacements of both Pb and Nb are aligned preferentially with the 〈111〉 directions and occur cooperatively over the nanoscale, forming polar 3D clusters. The arrangement of these clusters is nonrandom, resulting in aggregate PNRs that incorporate several inequivalent 〈111〉 clusters. The local alignment of displacements in such PNRs is maintained over an extended range, but the preferred alignment direction varies within a given region. The displacement magnitudes are largest near the PNR centers and decrease monotonically toward their boundaries, with a smooth transition from one PNR to another. No disordered non-polar matrix could be identified. The magnitudes of the Pb displacements are enhanced as the local Mg/Nb ratio increases; however, no spatial correlations between the PNRs and the much smaller CORs could be observed.

## Results

### Reconstructing the nanoscale structure from the diffraction data

We modeled the PMN structure at 300 K and 200 K using atomic configurations of 40 × 40 × 40 perovskite unit cells (320,000 atoms, 16  × 16  × 16 nm), which sample interatomic distances up to 8 nm (see the Methods section). The atomic coordinates in these configurations were varied according to an RMC algorithm^24,25^ while simultaneously fitting the neutron and X-ray powder total-scattering functions; their respective real-space Fourier transforms, which represent atomic pair-distribution functions (PDF); and 3D distributions of X-ray diffuse intensity measured using a single crystal. In addition, we included the extended X-ray absorption fine structure (EXAFS) data for Pb and Nb to provide chemical resolution. The refinements were enabled by several breakthrough developments in the RMC software and methodology which permitted efficient treatment of large atomic configurations, accurate fitting of the total-scattering data to long distances, and fitting of 3D diffuse-intensity distributions over an extended range of reciprocal space (see Methods).

**Fig. 1:**
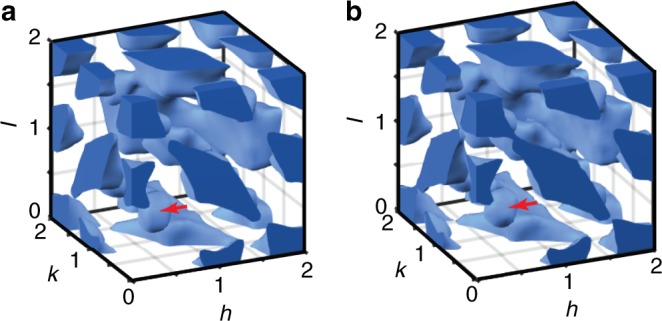
X-ray diffuse scattering. **a** Experimental and **b** calculated 3D constant-intensity surfaces for the X-ray diffuse-scattering intensity for a PbMg_1/3_Nb_2/3_O_3_ single crystal at 300 K. The dark shade of blue marks the intersections of these surfaces with the bordering box. Note the distinct shape of the diffuse scattering at the *h*00-type and *hk*0-type locations. The 3D diffuse-intensity distribution was fitted simultaneously with the neutron and X-ray total scattering and extended X-ray absorption fine structure data, as summarized in Supplementary Fig. 1. The red arrows indicate the ½ ½ ½ -type peaks associated with the chemical ordering of Mg and Nb

A summary of the experimental and fitted signals at 300 K with satisfactory agreement observed for all the data sets is displayed in Supplementary Fig. 1. Importantly, both neutron and X-ray PDFs are reproduced over the entire distance ranges and the calculated 3D diffuse scattering (Fig. 1; Supplementary Fig. 2) provides a close match to the experimental data. Not only the much-studied characteristic shapes near the Bragg reflections (i.e., butterfly for *h*00 and ellipsoidal for *hh*0) are reproduced but also the weak diffuse peaks at ½*hkl* (*h* = 2*n* + 1, *k* = 2*n* + 1, *l* = 2*n*) positions (Supplementary Fig. 3) and the overall *Q*-dependence of the diffuse intensity (Supplementary Fig. 4). Similar high-quality fits were obtained for 200 K.

### Chemical ordering of Mg and Nb

**Fig. 2:**
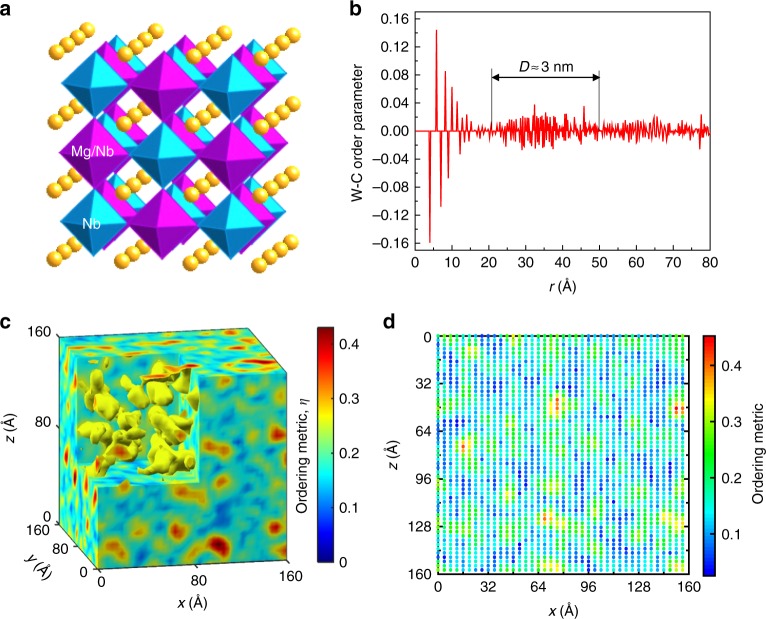
Characteristics of the chemical ordering of Mg and Nb. **a** A schematic rendering of the rocksalt-type partial ordering of Nb and Mg, with the alternate {111} planes occupied by Nb (blue octahedra) and the Mg_2/3_Nb_1/3_ (magenta octahedra) mixture, respectively. The Pb atoms are represented using yellow spheres. **b** The Warren–Cowley (W-C) short-range order parameter^30^ for the distribution of Mg and Nb, calculated as a function of interatomic distance for the refined configuration. A characteristic length, *D*, that corresponds to the effective size of chemically ordered regions is indicated. **c** A 3D distribution of the local chemical short-range order parameter, *η* (see the Methods section), which varies continuously across the regions of enhanced order. **d** A 2D map of the local order parameter calculated from the projected *Z*-map (see text) for the refined configuration as described in ref. ^27^. The *x* and *z* axes correspond to the [001] and [110] directions, respectively. This map agrees well with its analog derived from the experimental scanning transmission electron microscopy images^26,27^

While the existence of CORs, 2 –6 nm in size, can be regarded as well established, the details of the ordering, which influences the random electric fields and, in turn, the polar displacements^26^, are less certain. Recent results from atomic-resolution imaging in a scanning transmission electron microscope (STEM)^27,28^ and from 3D imaging using X-ray resonant scattering^29^ both support a partially ordered NaCl-type 1:1 arrangement with alternate {111} planes occupied preferentially by Nb and a 2:1 Mg/Nb mixture, respectively (Fig. 2a). From the STEM images^28^, the CORs appeared to exhibit a smoothly varying order parameter, being separated by wide anti-phase boundaries, rather than by a disordered matrix.

In reciprocal space, the cation ordering is manifested in the diffuse peaks at ½*hkl* (*h* = 2*n* + 1, *k* = *2n* + 1, *l* = 2*n* + 1) that are prominent in the single-crystal diffuse-scattering patterns (Fig. 1). We started with a random distribution of Mg and Nb on the octahedral sites and fitted these 3D peaks while swapping the locations of the two cations according to the RMC procedure. No atomic moves other than the Mg/Nb swaps were allowed at this stage. The distance dependence of the Warren–Cowley short-range order parameter^30^ calculated for the refined configuration (Fig. 2b) confirms the rocksalt-type ordering and suggests the presence of CORs, ≈2 –3 nm in size, without a disordered matrix. The order parameter for the first coordination sphere is at ≈70% of its maximum possible value. Similar results were obtained by starting with a rocksalt-type ordered arrangement of Nb and Mg instead of their random distribution.

We used a local short-range ordering metric, *η*, calculated similarly as described in ref. ^28^, to visualize a spatial distribution of the ordered regions. A 3D map of this metric for the refined configuration (Fig. 2c) reveals the CORs, which appear as the quenched spatial fluctuations of the order parameter, while the degree of ordering varied continuously across the system. We compared our results with those inferred from the experimental STEM images by calculating a (110) projection of our configuration box, with the projected atomic columns assigned intensities according to their average atomic numbers (*Z*) squared. The resulting image, which we call a *Z*-map, approximates the *Z*-contrast in STEM high-angle annular dark-field images (Supplementary Fig. 5a). The Fast Fourier transform (FFT) of this *Z*-map yields the ½½½-type superlattice diffuse spots like those obtained experimentally. Comparison of the calculated map and the experimental image, both FFT-filtered using these spots, demonstrates their qualitative agreement (Supplementary Fig. 5). Moreover, a map of the 2D ordering metric (Fig. 2d), calculated exactly as suggested in ref. ^28^, closely matches a similar map obtained by these authors from the experimental images (the thickness of their TEM samples was comparable with the dimension of our configuration box along the projection direction). Therefore, we concluded that the presently obtained models accurately represent the state of ordering in PMN and have adopted them for further refinements of atomic displacements (performed without any swap moves); the same Mg/Nb distribution was used for both 300 K and 200 K since the metal-ion diffusion at these temperatures is negligible.

### Cation displacements and PNRs

**Fig. 3:**
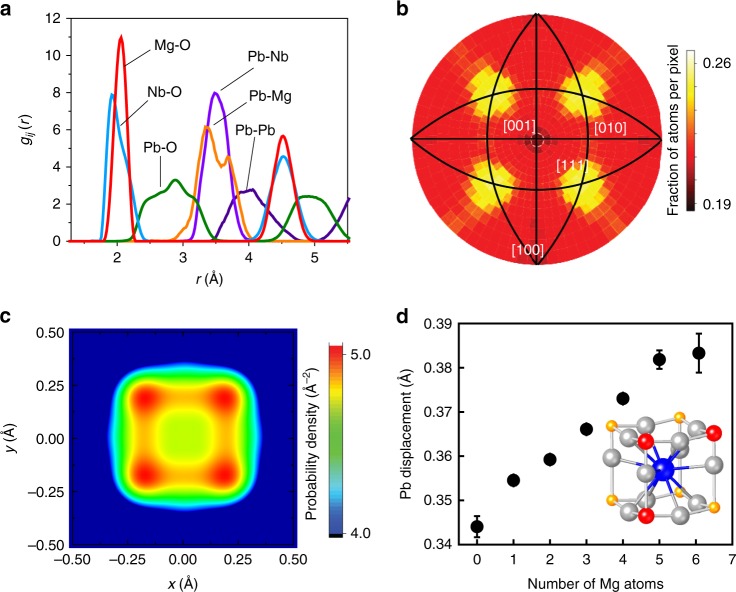
Characteristics of the cation displacements. **a** Key partial pair-distribution functions for the refined PbMg_1/3_Nb_2/3_O_3_ configuration. The Nb–O and Pb–O peaks are split, suggesting off-centering of the respective cations. In contrast, the Mg–O distribution exhibits a single symmetric peak. **b** A stereographic-projection map of the Pb displacements demonstrating their preference for the 〈111〉 directions. (The black lines indicate traces of the {110} planes). **c** A slice (at *z* ≈ 0.17 Å) of the probability density distribution for the Pb atoms revealing the four maxima offset along the 〈111〉 directions. **d** Magnitude of the Pb displacements as a function of the local Mg/Nb ratio around Pb. The inset illustrates the [PbO_12_(Mg/Nb)_8_] coordination. Pb, O, Mg, and Nb species are represented by the blue, light gray, red, and yellow spheres, respectively. The error bars represent single standard deviations estimated by analyzing three independently refined configurations

Partial metal–oxygen PDFs (Fig. 3a) calculated from the refined atomic coordinates confirm that both Pb and Nb are off-centered within the respective oxygen coordination polyhedra (cuboctahedra for Pb and octahedra for Nb), while Mg remains approximately central. The double-peak Pb–Mg and single-peak Pb–Nb distributions indicate much stronger off-centering of Pb relative to its Mg neighbors as compared with Nb, which is in line with the recent inferences from quantitative STEM imaging^27^. On average, all the cations retain their ideal cubic positions. Locally, however, both Pb and Nb atoms are displaced preferentially along the 〈111〉 directions, and this preference becomes more pronounced on cooling from 300 K to 200 K (Fig. 3b; Supplementary Fig. 6); the displacements of Mg appear to be isotropic. The probability density distribution (PDD) of Pb features eight well-separated 〈111〉 maxima (Fig. 3c) with the Pb atoms offset by ≈0.3 Å from the ideal cubic position. In contrast, the PDDs of Nb and Mg appear as a single peak. The Nb PDD exhibits a flattened top, which suggests either a flat potential well or an unresolved Nb-site splitting, whereas the Mg distribution is Gaussian. The root-mean-square displacements of Nb and Mg are ≈0.19 Å and ≈0.11 Å, respectively.

The observed off-center displacements of Pb^2+^ and Nb^5+^ agree with the known tendencies of these cations to form short, strongly covalent bonds with oxygen via hybridization of their respective 6s and 4d states with O 2p states. The magnitudes of the local Pb displacements increase with the Mg/Nb ratio in the [PbNb_8-*n*_Mg_*n*_] clusters (Fig. 3d), with a similar dependence obtained for both 300 K and 200 K. This trend can be attributed to the lower ionic charge of Mg and hence its less covalent bond with oxygen, which results in increasingly underbonded-oxygen atoms as *n* increases; therefore, larger Pb shifts are required to satisfy the oxygen-bonding requirements. Concurrently, Mg^2+^ has a larger ionic radius (≈0.70 Å) than Nb^5+^ (≈0.64 Å)^31^ resulting in the larger size of the cuboctahedral cages for the Mg-rich configurations; this ionic-radii difference is comparable to the difference in the values of Pb displacements for *n* = 0 and *n* = 7 (Fig. 3d).

The underbonded-oxygen argument also applies to the effects of Pb off-centering on the ordering of Mg and Nb. This off-centering, which modifies the bonding state of oxygen, has been previously suggested to stabilize the Mg/Nb configurations that could be electrostatically less favorable^32^. In fact, competition between long-range electrostatic and short-range Pb–O interactions could be a reason for favoring a partially ordered 1:1 type array of Mg and Nb over the completely ordered 1:2 layered arrangement. Thus, the existence of a relationship between the degree of Mg/Nb ordering and at least the static Pb displacements is chemically reasonable. We do observe that the overall Pb displacements in the instantaneous configuration are enhanced in the cores of CORs if only slightly; the small magnitude of this effect is determined by the continuously varying degree of ordering (see Supplementary Fig. 7 for a detailed analysis).

**Fig. 4:**
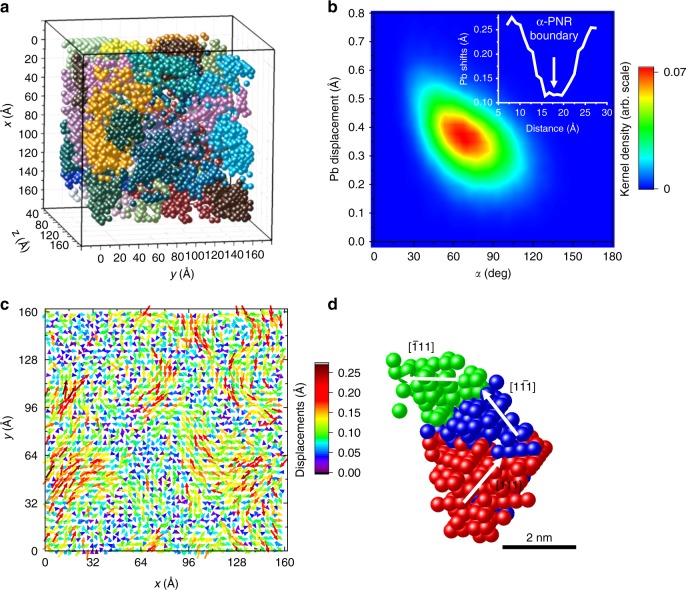
Correlations among the Pb displacements. **a** Clusters of Pb atoms having a local displacement-alignment metric *α* (see text) for the first Pb–Pb coordination sphere less than 45° (spatially distinct clusters are labeled using different colors). As a reference, *α* = 0° and *α* = 180° correspond to the parallel and antiparallel displacements of a given atom and its neighbors, respectively. The clusters have been identified according to the HDBSCAN^33^ algorithm. The search procedure accounted for the periodic boundary conditions; however, for visualization purposes, we assigned a distinct color to each region within the configuration box. **b** Magnitudes of the Pb displacements plotted against *α*; a strong negative correlation between the two characteristics is observed. The inset illustrates variation of the displacement magnitude for the Pb columns across the *α*-PNR boundary, as derived from panel **c**. **c** A 2D map of displacements for the Pb columns projected onto a {110} plane; the color-scale bar reflects the displacement magnitude in Å and the arrows are used to illustrate the displacement directions. The *x* and *y* axes correspond to the orthogonal [110] and [001] directions. This distribution agrees with the analogous maps deduced from experimental atomic-resolution scanning transmission electron microscopy images of PbMg_1/3_Nb_2/3_O_3_. The displacements decay monotonically toward the boundaries. **d** 3D rendering of a representative *α*-PNR, with the constituent Pb atoms (represented by spheres) that are displaced along the inequivalent 〈111〉 directions (labeled in the figure) indicated using different colors

The 3D Pb-displacement correlations in the form of dense nanoscale regions are evident from considering the local alignment metric, *α*, defined as the average of the angles between the displacement vectors of a given atom and each of its neighbors within a sphere of a certain radius. Figure 4a displays the Pb atoms with *α* < 45° (for the first Pb–Pb coordination shell) that have been identified by the HDBSCAN algorithm^33^ to form dense, spatially distinct clusters (labeled using different colors). In this representation, PMN appears as an assemblage of regions, about 4–6 nm in size, featuring the locally aligned displacements; we will call these regions *α*-PNRs. The displacements of Nb are strongly and positively correlated with those of Pb (Supplementary Fig. 8). Presumably, these correlations are driven by the bonding requirements of the oxygen atoms.

The values of *α* are minimal near the centers of *α*-PNRs and increase gradually toward their boundaries. The magnitude of the Pb displacements scales inversely with *α* (Fig. 4b). Overall this picture agrees with the results of the recent MD studies^17^, which suggested a high incidence of domain walls without a disordered matrix. Projections of the Pb-displacement field onto the {110} planes also agree with the displacement patterns identified in the recently published STEM images^27^ (Fig. 4c). Figure 4b (inset) illustrates smooth variation of the displacement magnitude across the *α*-PNR boundary. Our refinements at 200 K, which is below the freezing temperature *T*_f_≈230 K, reveal significant growth of the *α*-PNRs with a concurrent increase in the magnitudes of the Pb displacements (Supplementary Fig. 9). This observation agrees with the previously reported experimental and modeling data on the temperature behavior of PMN. Also, the estimated sizes of *α*-PNRs are comparable with the correlation lengths identified in earlier studies using parameterized fits of variable-temperature neutron PDFs^9^.

The definition of PNRs according to the local alignment angle disregards displacement directions. We added this directional specificity by identifying those Pb atoms that have their displacement vectors aligned with the 〈111〉 directions. Pb atoms displaced along a given 〈111〉 direction also form extended 3D clusters, which we will refer to as 〈111〉-PNRs. The 〈111〉-PNRs and *α*-PNRs have comparable sizes, however, the correlation length of the displacement magnitudes within the 〈111〉-PNRs is considerably shorter than that of the displacement directions.

Typical *α*-PNRs are composed of portions of 2–3 〈111〉-PNRs, which are arranged so that the adjacent regions are preferentially 71° 〈111〉 variants (Fig. 4d; Supplementary Fig. 10). This preference, which also agrees with the MD simulations^17^, is evident both from the analysis of the individual *α*-PNRs and from the histograms that describe distributions of distances between the Pb atoms that belong to the 71°, 109°, and 180° variants, respectively (Supplementary Fig. 11). Thus, PMN exhibits a hierarchical PNR structure, with the correlated arrangement of the 〈111〉-PNRs yielding the aggregate *α*-PNRs; this aggregate nature of *α*-PNRs is retained even if the threshold value of *α* used to identify them is reduced. No relation could be found between the PNRs and the significantly smaller CORs; the size difference between these two types of regions becomes particularly pronounced at 200 K.

Diffuse-scattering simulations using only those Pb atoms that belong to a single variant (out of 8) of the 〈111〉-PNRs could not reproduce the characteristic shapes of the diffuse scattering, which emerged only in the presence of all the variants. This result resonates with the assessment by Takenaka et al.^17^, who attributed the diffuse scattering in PMN to a multidomain state. We investigated the nature of such a multi-domain state by performing an inverse Fourier transform (IFT) of the calculated diffuse-scattering amplitude around the Bragg peaks (spherical masks 0.2 Å^−1^ in diameter were used). Extensive testing using simulated structures demonstrated that IFT successfully recovers regions of correlated displacements and the displacement patterns that underlie specific diffuse-scattering features. The Pb atoms that are associated with the largest variation of the amplitude of the IFT (see Methods) form 3D clusters each composed predominantly of the two 71° 〈111〉-PNRs with nearly all the Pb atoms having *α* < 70° (Supplementary Fig. 12); a spatial distribution of these regions resembles that of *α*-PNRs. For an aggregate of the two equal-volume 71°〈111〉-PNRs, the effective polarization direction is 〈110〉, which is consistent with the anisotropy of the diffuse-scattering distributions. Thus, the much-studied diffuse-scattering features appear to arise from *α*-PNRs, which develop because of the correlated spatial arrangements of 〈111〉-PNRs coupled with concurrent modulation of the displacement-magnitudes.

We also applied IFT to the calculated diffuse-scattering peaks at the ½½0-type locations (Supplementary Fig. 3), the so-called M-points, which previously have been attributed to local ordering of antiparallel cation displacements that signify antiferroelectric interactions^18,34^. The analysis of these weak peaks is complicated because they reside on a large background and overlap with the diffuse-intensity tails that extend from the Bragg reflections (ref. ^34^; Supplementary Fig. 13). Nevertheless, by zooming in on the locations of the Pb atoms that exhibit the largest variation of the IFT amplitude associated with the M-points, we could identify the nanoscale antiparallel ordering of the 〈100〉 Pb-displacement components (depicted schematically in Supplementary Fig. 14). This ordering differs from the model with antiparallel 〈110〉 Pb displacements proposed in ref. ^34^; however, we could not discriminate the background effects sufficiently to visualize the entire population of such ordered clusters responsible for the M-points and, therefore, the presence of several types of displacement patterns cannot be ruled out.

### Coupling of cation displacements to the oxygen framework

Concerted cation displacements in the PNRs are expected to be accompanied by characteristic distortions of oxygen octahedra. We analyzed local distortions of the octahedral framework by expanding oxygen displacements for the individual octahedra into a set of deformation modes, and then searched for correlations among these modes and the cation displacements. A full orthonormal basis of the 18 deformation modes for an octahedron (Supplementary Fig. 15) was defined to reflect common lattice distortions (three tetragonal, three orthorhombic, and three octahedral rotations), with the remaining modes (three translational, six bending, F_1u_- and F_2u_) selected to provide a complete set. In this basis, a symmetric octahedron’s breathing mode (A_1g_) is the sum of the three tetragonal modes, whereas the rhombohedral deformations can be calculated from the combinations of the orthorhombic and tetragonal modes. The amplitudes of the 18 basis modes were determined for every octahedron in the configuration.

**Fig. 5:**
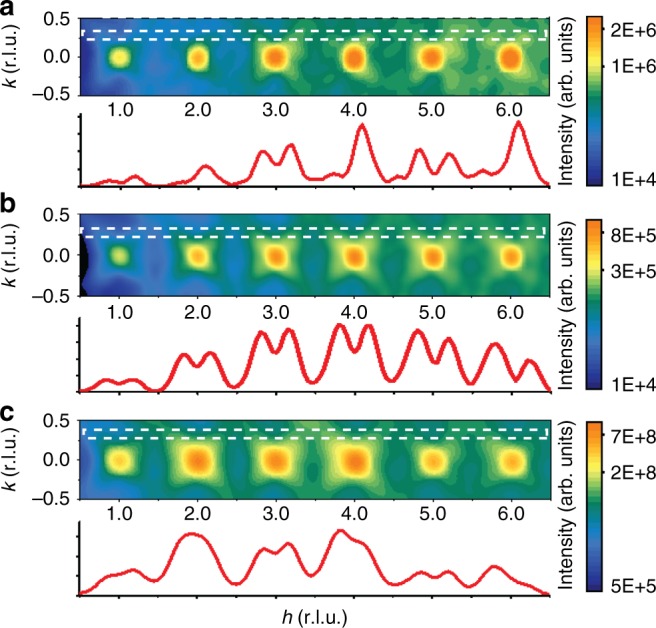
Simulated neutron diffuse scattering. **a**, **b** Neutron diffuse scattering near the *h*00 reflections (*h* is the horizontal axis) calculated for the refined configuration at 300 K with (**a**) all the species and (**b**) only the Pb atoms included in the calculations. **c** X-ray diffuse scattering near the same reflections with all the species included in the calculations. The intensity traces correspond to the *k*-positions indicated using white/dotted rectangles; the logarithmic intensity scales are the same as indicated in the corresponding color-scale bars. The neutron scattering in (**a**) is modulated relative to (**b**), featuring different appearance for the asymmetry of the *h*00 reflections with *h* = 2*n* and *h* = 2*n* + 1. This modulation, which is associated with the correlated oxygen displacements, reproduces the experimental results reported in ref. ^18^, even though the present refinements did not include neutron diffuse scattering

Only the translational and the bending F_1u_ (Supplementary Fig. 15) modes appeared to be coupled to the directions of the cation displacements. The Pb displacements are negatively correlated with those of oxygen in these modes for both the [Pb_8_(NbO_6_)] and [Pb_8_(MgO_6_)] clusters. Similar negative correlations are observed for the Nb displacements, whereas for Mg the correlations are strongly positive. The resulting oxygen-cation displacement patterns (Supplementary Fig. 16) match the low-frequency transverse optical modes commonly encountered in PMN^35^ and other perovskite ferroelectrics, which indicates that even such complex multiatomic correlations are reproduced in the refined configurations. Similar trends hold for the *x*, *y*, and *z*-axis components of the deformation modes and cation displacements. No dependence of the deformation-mode amplitudes and the cation-oxygen coupling on the chemical order parameter was observed. As a soundness check for the procedure, the mean amplitudes of the breathing modes for the [NbO_6_] and [MgO_6_] octahedra were found to be negative and positive, respectively, which reflects oxygen displacements away from Mg toward Nb, consistent with the Mg^2+^–Nb^5+^ ionic charge difference and the stronger covalency of the Nb–O bonds; the values of the two amplitudes are correctly related according to the Nb/Mg stoichiometry. The observed correlated oxygen displacements account for a distinct appearance of the neutron single-crystal diffuse scattering near the *h*00 Bragg peaks with *h* = 2*n* and *h* = 2*n* + 1 (Fig. 5); the agreement with the experimental plots reported in ref. ^18^ is especially striking given that these data were not included in the present fits.

Atomic configurations obtained from X-ray and neutron-scattering data represent structural snapshots with superposition of both the static and dynamic displacements (see the Methods section for a discussion of the effects of energy integration on both types of data); the latter reflect contributions from acoustic and low-frequency optical phonons. Our observations of the negative correlations between the oxygen and Pb/Nb displacements indicate that the oxygen-shifts involved in such optical modes are sufficiently large to overcome the usually dominant effect of acoustic motion.

## Discussion

The combination of X-ray and neutron total-scattering data with a 3D distribution of diffuse-scattering intensity has proven to be effective for unraveling complex nanoscale perturbations of the average structure, such as encountered in PMN; our tests demonstrated that a complete picture could not be recovered using any of these techniques alone. The reconstructed chemical and displacive nanoscale ordering in PMN agrees well with the reported STEM images^27,28^, which attests to the fidelity of the present structural models and of the information that can be extracted from quantitative STEM measurements. The obtained atomistic picture invalidates the notion of chemical or polar-ordered regions dispersed in a disordered matrix, instead suggesting continuously varying order parameters. The displacements of Pb and Nb atoms at both 300 K and 200 K exhibit preference for the 〈111〉 directions, in contrast to previously proposed spherical-shell distributions. The 〈111〉 displacements are spatially correlated, but an additional higher-level correlation exists that is manifested in a preference for neighboring 〈111〉 clusters to represent 71° variants, resulting in a hierarchical assembly of PNRs; displacement-magnitudes follow this latter correlation. Preference for the 71° variants has also been noted in the most recent MD simulations of PMN^17^, which together with our results point to the significance of this effect. Consistent with the previously made inferences, the PNRs grow on cooling to 200 K. In addition, nanoscale regions with ordered antiparallel Pb displacements are shown to exist alongside the PNRs. While we observe no spatial coincidence between the PNRs and CORs, chemical ordering is still expected to affect the behavior of polar displacements, as can be inferred from the dependence of Pb displacements on the local Mg/Nb ratio. Our results suggest that the existing models of PMN that ignore the continuously varying degree of chemical ordering and overlook the inter-PNR correlations should be reconsidered. Understanding the origins of the inter-PNR correlation, its evolution with temperature and chemical substitutions, and ultimately its significance for the dielectric response represent topics for future theoretical and experimental research on PMN and its solid solutions. A logical step would be to perform similar refinements but add the 3D neutron diffuse scattering^18^ to the data suite, which should provide more accurate correlations of the oxygen displacements.

As a more general remark, we note that with recent advances in neutron and synchrotron instrumentation, measurements of 3D diffuse-intensity distributions over a broad range of reciprocal space have been facilitated, creating opportunities for wider use of the approach described in this study. The accuracy and scope of structural information that can be recovered using combined-technique refinements are largely limited by the quality of experimental data and the sizes of atomic configurations that can be handled within reasonable computing times. The currently used atomic configurations are still at the lower limit of what is required for accurate treatment of nanoscale correlations in systems like PMN. In fact, since the PNRs grow at 200 K, this configuration-size already becomes insufficient. Thus, harnessing the full power of nanoscale atomistic structural refinements requires improved instrumentation, more accurate data-reduction procedures, and efficient structure-refinement computer algorithms that would take full advantage of the underused computing resources available today.

## Methods

### Synthesis

A pyrochlore-free ceramic sample of PMN was prepared by solid-state reaction according to the columbite route proposed in ref. ^35^. Powders of MgO and Nb_2_O_5_ (with 2 mol% excess MgO) were reacted at 1000 °C for 8 h to form MgNb_2_O_6_. This product was batched with a stoichiometric amount of PbO and heated in a covered crucible at 800 °C for 4 h to form phase-pure PMN. Prior to each heating, the powders were mixed and ground in a planetary ball mill in isopropanol using yttria-stabilized zirconia grinding media.

### Total-scattering measurements

Neutron total-scattering data were collected at 300 K and 200 K using the Polaris diffractometer at the ISIS facility (Science and Technology Facility Council, UK). The sample powder was loaded in a vanadium can with temperature control achieved using a displex cryostat. The data were processed using the GUDRUN software to obtain the total-scattering function *S*(*Q*) and its Fourier transform, *G*(*r*) (*Q*_max_ = 35 Å^−1^). X-ray total scattering was measured at the 11-ID-B beamline of the Advanced Photon Source (Argonne National Laboratory) using an incident-beam energy of ≈60 keV with the area detector positioned at 18 cm downstream from the sample. The sample was loaded in a 1 -mm kapton capillary. The X-ray data were processed using the PDFGetX3 software to obtain the corresponding total-scattering function and its Fourier transform (*Q*_max_ = 25 Å^−1^). In both neutron and X-ray total-scattering measurements, the instrument resolution was characterized by measuring NIST Si SRM powder.

Extended X-ray absorption fine structure (EXAFS) measurements were performed for the Pb *L*_3_ and Nb *K* edges at the NIST beamline 06-BM at NSLS-II (Brookhaven National Laboratory). The data were collected at room temperature in transmission. All the data were processed using Athena^36^. Initial fitting was accomplished in the Artemis software^37^. Scattering amplitudes and phases were calculated using FEFF8^38^. For the Nb EXAFS, the *k*-space data were multiplied by *k*^2^ prior to the Fourier transform, which was performed over the *k*-range from 3.3 Å^−1^ to 13.2 Å^−1^; the *r*-space fitting range was from 1 Å to 4.2 Å, with both single- and multiple-scattering paths of a photoelectron included in the fit. For Pb, the *k*-space data were multiplied by *k* and the *k*-space range used in the transform extended from 2.2 Å^−1^ to 10 Å^−1^; the *r*-space fit was performed from 1 Å to 3.5 Å, with only the single-scattering paths included.

### Transmission electron microscopy

Transmission electron microscopy (TEM) was performed on the crushed powder dispersed on a lacey carbon-coated copper grid. Selected area electron diffraction patterns and high-angle annular dark-field images were collected at 300 kV in the TEM and scanning TEM modes, respectively.

### Single-crystal X-ray diffuse scattering

X-ray single-crystal scattering measurements were performed at the beamline ID29 of ESRF. A rod-like 50-μm-thick crystal was mounted on a rotation stage and held in a cryostream flux for temperature control. Prior to the measurements, the sample was etched using hot concentrated hydrochloric acid. Diffuse-scattering patterns were recorded with the wavelength of 0.954 Å over the angular range of 360° in 0.1° increments on a single-photon counting pixel detector, which allowed for the shutter-less data collection. The CrysAlis software package was used to refine the orientation matrix and the final reciprocal-space reconstructions of 3D diffuse-intensity distributions were accomplished with locally developed computer software.

### Structure refinements

Structural refinements were performed using the development version of the RMCProfile software^24^. This version makes extensive use of GPU computing (yet unavailable for the distribution package of RMCProfile), which is critical for achieving computational speeds required to handle large atomic configurations and calculations of 3D diffuse scattering involved in this study. The PMN structure was modeled using atomic configurations of 40 × 40 × 40 unit cells, which included 320,000 atoms (Mg/Nb ratio is equal to 0.49998828) and covered interatomic distances up to 8 nm. Neutron- and X-ray total-scattering data in both real and reciprocal spaces were fitted simultaneously with the two EXAFS data sets and the 3D X-ray single-crystal diffuse-intensity distribution; in addition, the neutron Bragg profile was included in the fit. The X-ray data were calculated considering the Q-dependence of the X-ray scattering cross-sections.

In both the neutron and X-ray cases, the calculated total-scattering data were corrected for the instrument resolution in reciprocal and real spaces prior to comparison with the corresponding experimental signals. The resolution corrections accounted for the actual peak profile shapes, which is especially important for the time-of-flight neutron data that exhibit significant peak asymmetry. The accuracy of these corrections is key for obtaining meaningful results while fitting a pair-distribution function to longer distances. The 3D X-ray diffuse scattering has been calculated according to the formalism outline by Butler and Welberry^39^. Prior to comparison with the experimental data, the calculated diffuse-scattering intensity was smoothed using a 3D Gaussian filter. The weights assigned to individual data sets, which control the contribution of a given data set to the total residual, were adjusted during fitting using a newly developed automated routine that ensures that all individual residual terms, while allowed to increase temporarily, over time decrease to small user-preset values. This procedure, which involves an analysis of statistical correlations between changes in the aggregate residual function and each of its terms, drastically accelerates convergence of RMC refinements.

For X-ray total scattering, the energy-integration range is always significantly broader than a span of phonon energies and therefore these data reflect instantaneous atomic positions. For EXAFS, a characteristic X-rays-sample interaction time is ≈10^−15^ s thus also probing an instantaneous PDF. In the case of neutron total scattering, the actual energy-integration range is limited by the so-called Placzek shift and reportedly can be less than 10 meV^40,41^. However, for a given material, the exact value of this limit will vary between different instruments and is somewhat uncertain^40^. According to previous studies of PMN using a dynamic PDF^41^, at *T* ≥ 450 K, the off-centering of Pb, which is manifested in the splitting of the nearest-neighbor Pb–O peak with the appearance of short Pb–O distances at ≈2.4 Å, is purely dynamic. Our variable-temperature X-ray PDF (X-PDF) data (Supplementary Fig. 17a) demonstrate that this splitting and the short Pb–O distances are observable at least up to 490 K, as expected since X-ray total scattering reveals both static and dynamic correlations. Our neutron PDF (N-PDF) at 490 K (Supplementary Fig. 17b) also features a clearly identifiable splitting of the Pb–O distribution with a peak at ≈2.4 Å, as confirmed by the presence of anomalies in the second derivative of the PDF signal (Supplementary Fig. 17c). Considering that the onset energy for a phonon that involves the off-centering of Pb is ≈15 meV^41,42^, we conclude that the integration range for our neutron total-scattering measurements extends at least past this energy, and thus the low-energy atomic vibrations are captured in the data.

### Analyses of refined atomic configurations

The 3D local cation-order metric used here to describe a distribution of Mg and Nb over the octahedral sites was defined by adopting an approach proposed in ref. ^16^ to characterize the Mg/Nb ordering from atomic-resolution STEM images of PMN. In this work, the order metric has been equated with the coefficient of variation of intensities for the Mg/Nb columns surrounding a Pb column. For a disordered case, all the Mg/Nb columns have similar average atomic numbers/intensities, and the coefficient of variation is zero. In contrast, strong ordering yields large values of this metric. In this case, we defined the ordering metric, *η*, as *η* = (*n*_1_ − *n*_2_)^2^ + (*n*_4_ − *n*_3_)^2^, where *n*_i_ is the fraction of, say, Nb atoms found in the *i*^th^ coordination shell around a given B-cation (Nb or Mg) normalized to the fraction expected for this shell from the average concentration of Nb. The metric was calculated for each B-cation in the configuration. With this definition, clusters of the B-cations with large values of *η* represent chemically ordered regions.

Inverse Fourier-transform filtering of the complex diffuse-scattering amplitude calculated from the refined atomic coordinates was performed by placing spherical masks with diameter 0.2 Å^−1^ around the features of interest. The amplitude of this transform was calculated around each Pb atom on a grid with a mesh size of 0.05 Å. The atoms that provide the largest contribution to the diffuse scattering features used in the inverse Fourier transform are associated with the largest difference between the maximum and minimum amplitudes. The distance vector that corresponds to the spatial locations of these extrema reflects a component of the atomic displacement that participates in the correlations yielding the diffuse feature of interest.

Distortions of the oxygen octahedral framework from an ideal configuration were characterized by introducing a set of deformation modes for the oxygen octahedra. For a more intuitive interpretation, the modes were selected to mimic both typical lattice distortions (e.g., tetragonal, orthorhombic) and vibrational modes (rotations, bending) while providing a complete orthonormal basis. The resulting modes are depicted in Supplementary Fig. 15, whereas their corresponding coordinate-transformation matrices for cubic symmetry are summarized in Supplementary Table 1. All the modes, except for the tetragonal, preserve an octahedron’s volume. The orthorhombic and rotational modes describe oxygen coordinates adequately within the limits of the small-angle (linear) approximation.

## Supplementary information

Supplementary Information

## Data Availability

The experimental data that support the findings of this study are available from the corresponding author upon reasonable request. A file with a representative refined atomic configuration is provided as a part of Supplementary Information, 10.6084/m9.figshare.7870955.

## Code availability

The RMCProfile software (executables) can be downloaded from www.rmcprofile.org.
