# Supplementary material for: Local atomic order and hierarchical polar nanoregions in a classical relaxor ferroelectric
Source: Nat Commun. 2019 Jun 21;10:2728. doi: 10.1038/s41467-019-10665-4 (PMC6588601; doi:10.1038/s41467-019-10665-4)
Supplement: Supplementary file 1 — Supplementary Information [file 41467_2019_10665_MOESM1_ESM.pdf]

Supplementary Information

Local Atomic Order and Hierarchical Polar Nanoregions in a Classical Relaxor Ferroelectric

Eremenko et al.

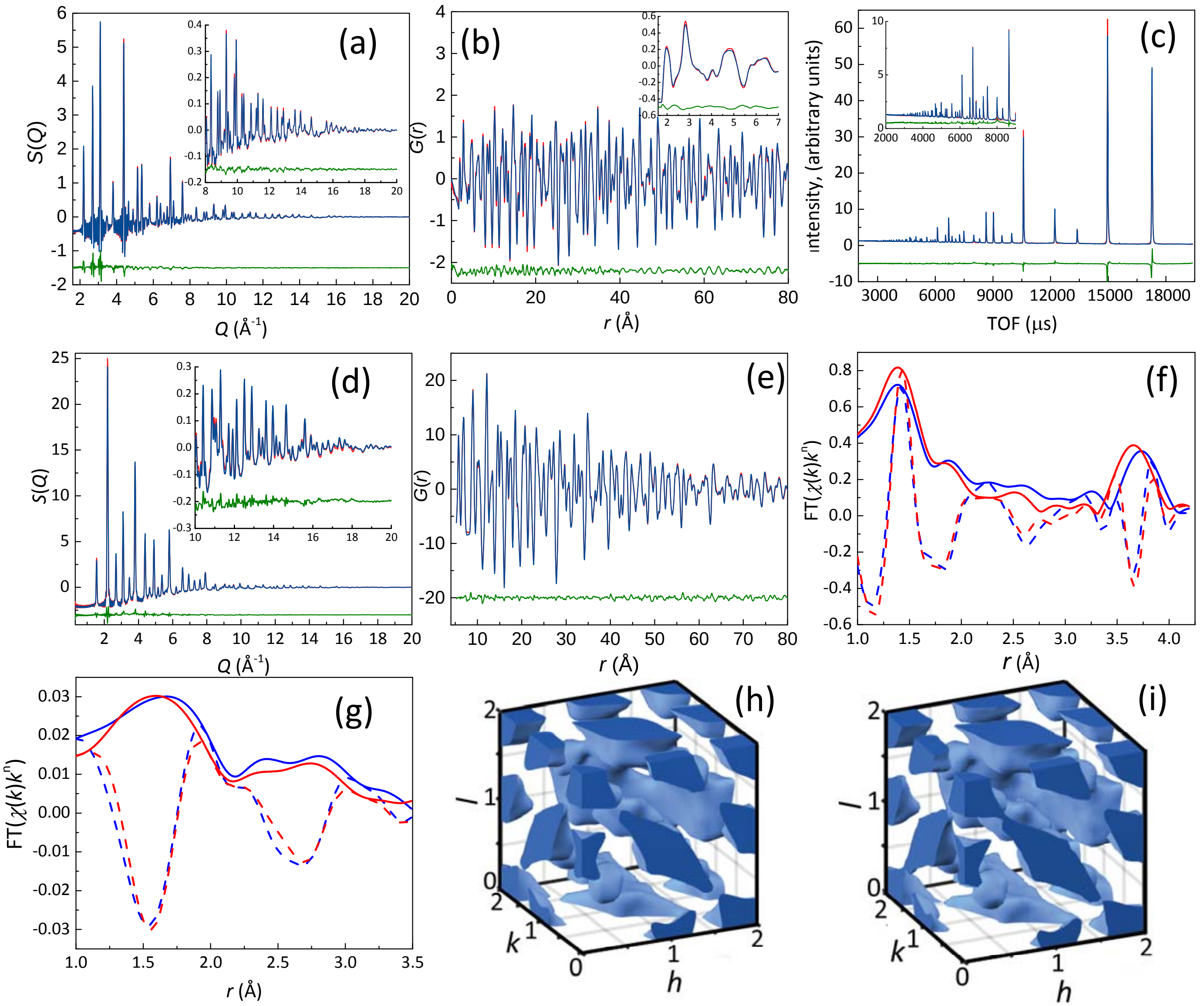

Supplementary Figure 1: Results of data fitting for $PbMg_{1/3}Nb_{2/3}O_3$ at 300 K. (a-g) Experimental (red) and calculated (blue) signals: (a, b) Neutron total-scattering function, $S(Q)$, and its Fourier transform, $G(r)$, respectively; (c) Neutron Bragg profile; (d-e) X-ray total scattering function and its Fourier transform, respectively, (f) Nb EXAFS, (g) Pb EXAFS. (h-i) Experimental and calculated constant-intensity surfaces for the X-ray diffuse intensity, respectively. In (a-e) the residual curves are colored in green. For insets, the axes labels and scales are the same as for the main plots. For the EXAFS fits, the magnitude (solid line) and the imaginary part (dashed line) of the Fourier transform are shown. The calculated signals correspond to a configuration refined using simultaneous fitting of all the experimental data with a single atomistic model. Note: Strong ripples around Bragg peaks in the neutron $S(Q)$ result from the convolution of the experimental data with a box function, which accounts for a finite size of the atomic configuration, prior to comparison with the calculated $S(Q)$ signal. In the X-ray case, these ripples are less noticeable because X-ray Bragg peaks in the present experimental data are considerably broader than their neutron analogs. The residual values for the fitted datasets are as following: neutron S(Q) – 0.67 %, neutron $G(r)$ – 0.60 %, neutron Bragg – 0.56 %, X-ray $S(Q)$ – 0.26 %, X-ray $G(r)$ – 0.26 %, EXAFS Nb – 9.6 %, EXAFS Pb – 8.9 %, X-ray diffuse – 3.6%.

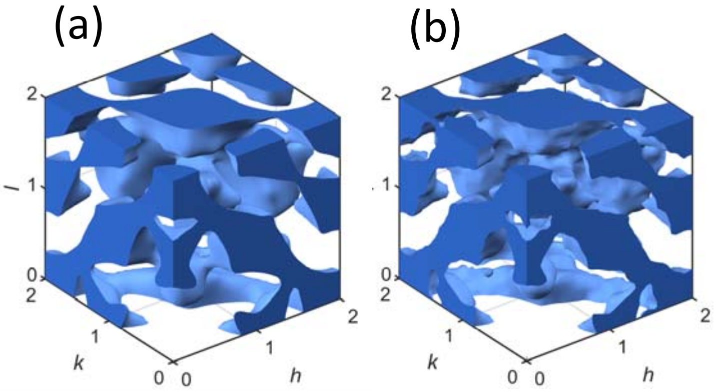

Supplementary Figure 2: 3D X-ray diffuse scattering intensity distributions. Experimental (a) and calculated (b) constant-intensity surfaces for the X-ray diffuse intensity at 200 K.

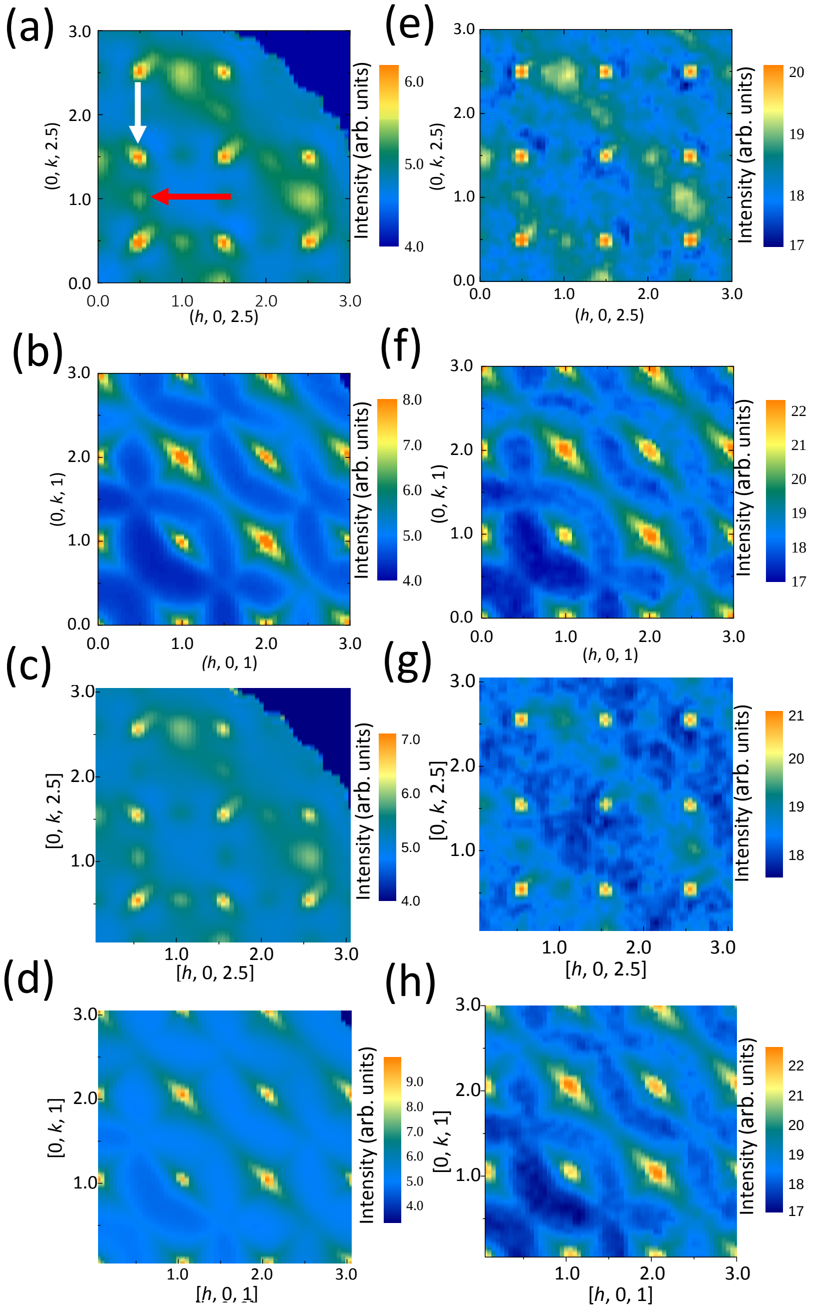

Supplementary Figure 3: Sections of the X-ray diffuse-scattering intensity. Experimental (a-d) and calculated (e-h) *hk*2.5 (a, c, e, g) and *hk*1 (b, d, f, h) sections of the diffuse X-ray scattering intensity distribution at 300 K (a, b, e, f) and 200 K (c, d, g, h). The experimental data are reproduced even beyond the fitting range of *h*=(0:2), *k*=(0:2), *l*=(0:2). The intensity peaks at the ½½½-type and ½½0-type locations are indicated using the white vertical and red horizontal arrows, respectively.

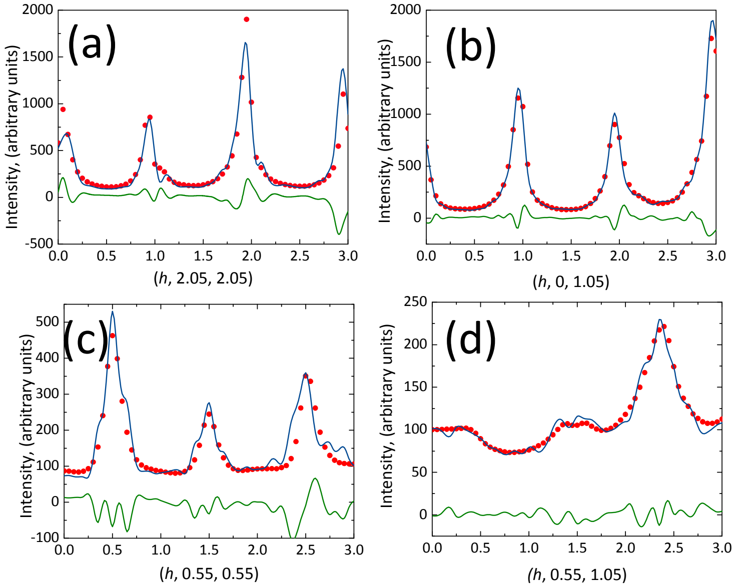

Supplementary Figure 4: Experimental (red) and calculated (blue) profiles of the diffuse X-ray scattering intensity at 300 K along several representative directions. These directions are (a) [*h*,2.05,2.05], (b) [*h*,0,1.05], (c) [*h*,0.55,0.55], (d) [*h,*0.55,1.05]. The residual curves are colored in green. These plots demonstrate that the *Q*-dependence of the diffuse scattering is reproduced.

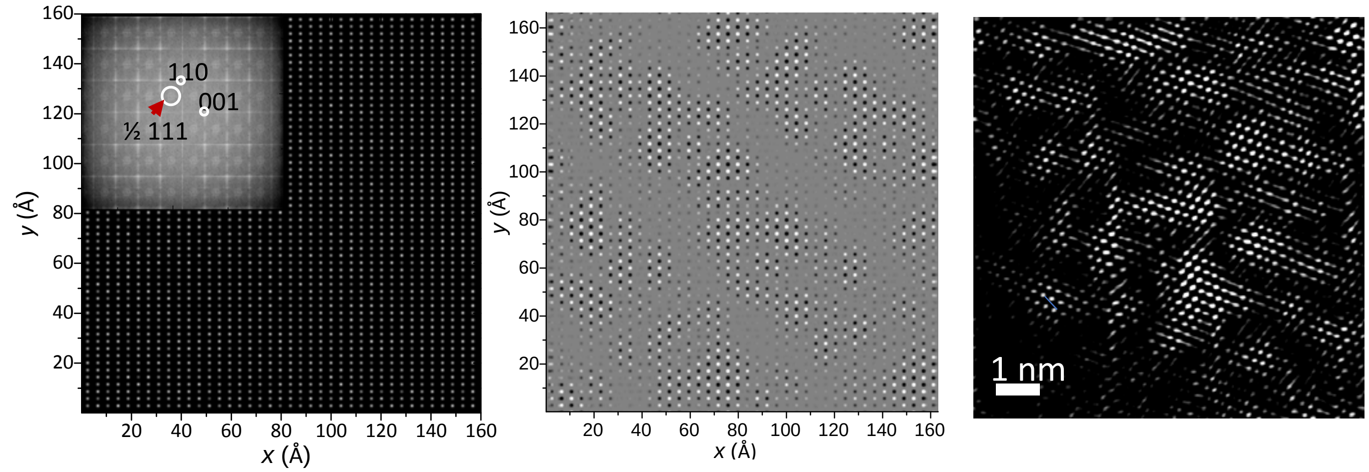

(a) (b) (c)

Supplementary Figure 5: Comparison with scanning transmission electron microscopy images. (a) A {110} projection of the refined configuration with the atomic columns assigned intensities according to Z^2, where Z is the average atomic number for a column. Only the columns occupied by Mg and Nb are displayed. The Fast Fourier Transform (FFT) of this image (inset) reveals diffuse ½ ½ ½-type spots (indicated using a red arrow) characteristic of rocksalt-type ordering (like the diffuse spots indicated by the red arrow in Fig. 1 of the main text). (b) The inverse FFT using the diffuse spots only, which highlights the regions with strong chemical order. (c) similarly filtered experimental STEM image of PMN. Qualitative agreement of (b) and (c) is evident.

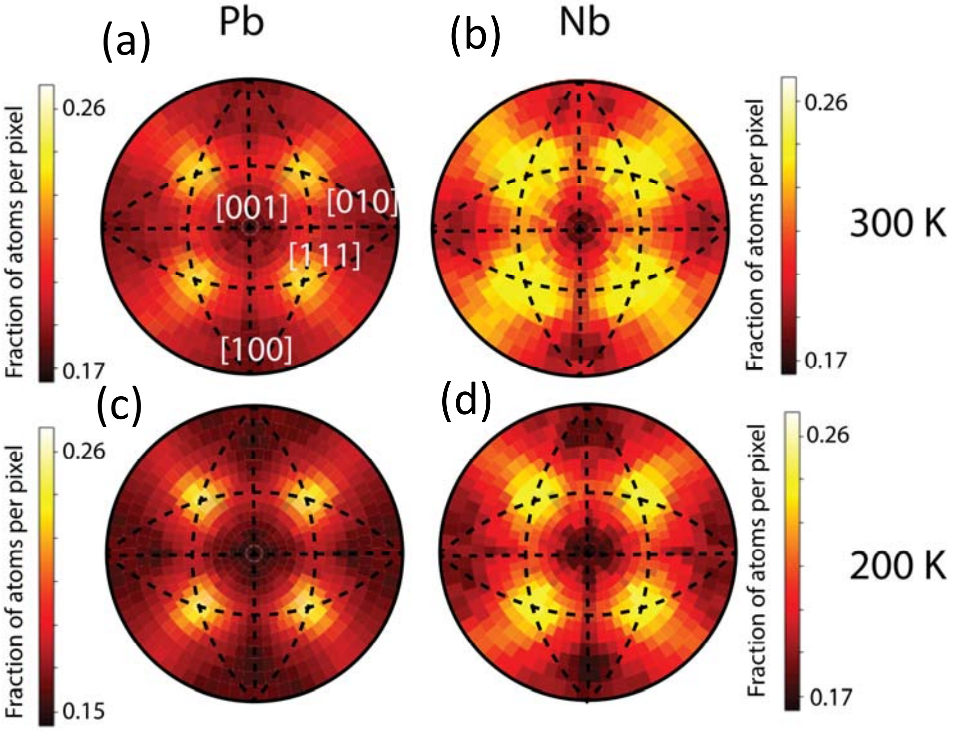

Supplementary Figure 6: Maps of the cation-displacement directions. Stereographic-projection maps of the displacement directions for Pb (a, c) and Nb (b, d) at 300 K (a, b) and 200 K (c, d).

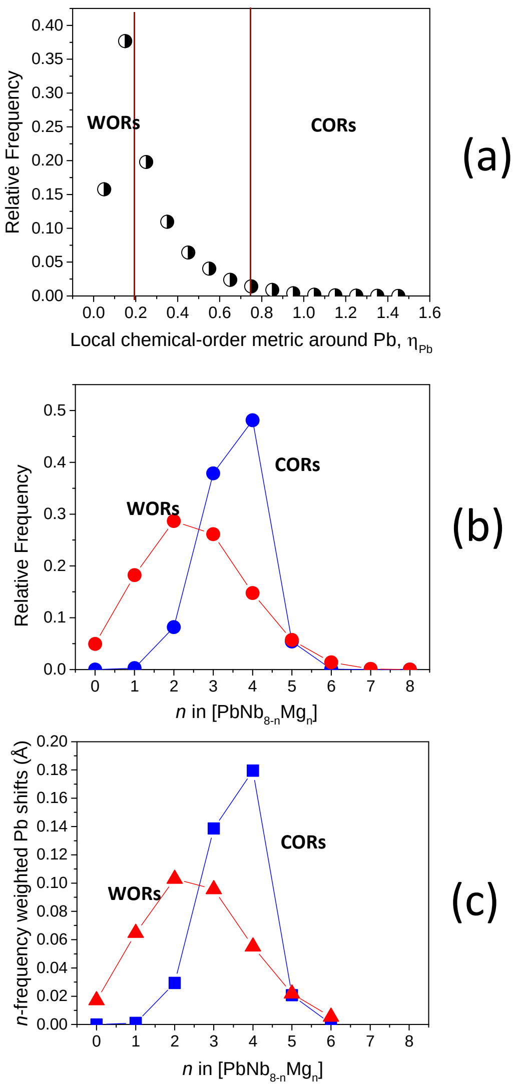

Supplementary Figure 7: Chemical Mg/Nb ordering and Pb displacements. (a) A statistical distribution of the local Mg/Nb ordering metric, η_{Pb}. This metric was calculated for each Pb atom in the configuration as an average of the η-values (see Methods) for its 8 nearest-neighbor B-cations (Nb and Mg). Parts of the η_{Pb} distribution with η_{Pb} <0.2 and η_{Pb} >0.8 were ascribed to the weakly (WORs) and strongly (CORs) ordered regions, respectively. (b) Statistical distributions of the number of Mg atoms, *n*, in the $[PbNb_{1-n}Mg_n]$ coordination clusters for Pb atoms within WORs and CORs; These two distributions are expectedly different. The distribution for the CORs is significantly narrower and peaks at *n*=4 as opposed to *n*=2 for Pb atoms in the weakly ordered regions (WORs). (c) The relative frequencies for the *n*-values in (b) were multiplied by the corresponding magnitudes of Pb displacements (extracted from Fig. 3d, main text) to calculate *n*-frequency-weighted Pb displacements within the CORs and WORs, respectively, which are displayed as a function of *n*. Note the similarity between (b) and (c). From (c), the resulting values of Pb displacements for each type of region were calculated: ≈0.37 Å (CORs) and ≈0.36 Å (WORs). This difference albeit small is statistically significant. Evidently, its small value is determined by the statistical distributions of the *n*-values in regions with different degrees of cation ordering which, in turn, reflect the state of ordering in the system.

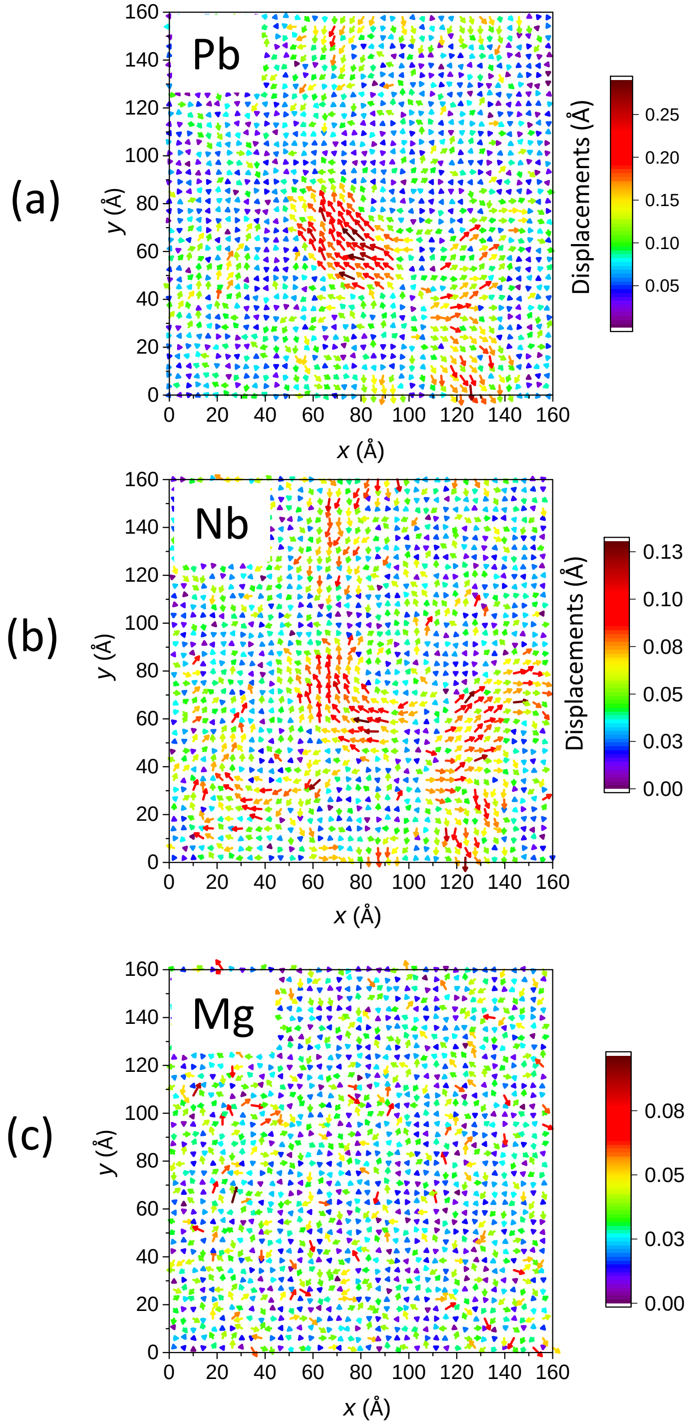

Supplementary Figure 8: Maps of cation-column displacements. Maps of displacements of the (a) Pb, (b) Nb, and (c) Mg columns at 300 K projected onto a {100} plane; the axes *x* and *y* correspond to the orthogonal ⟨100⟩-type directions. The vector lengths for the Nb and Mg column displacements are scaled relative to those for Pb by the factors of ×2 and ×3, respectively. The displacements of the Pb and Nb columns are strongly correlated, forming clusters of aligned displacements at the same locations. No such correlations are observable for the Mg columns.

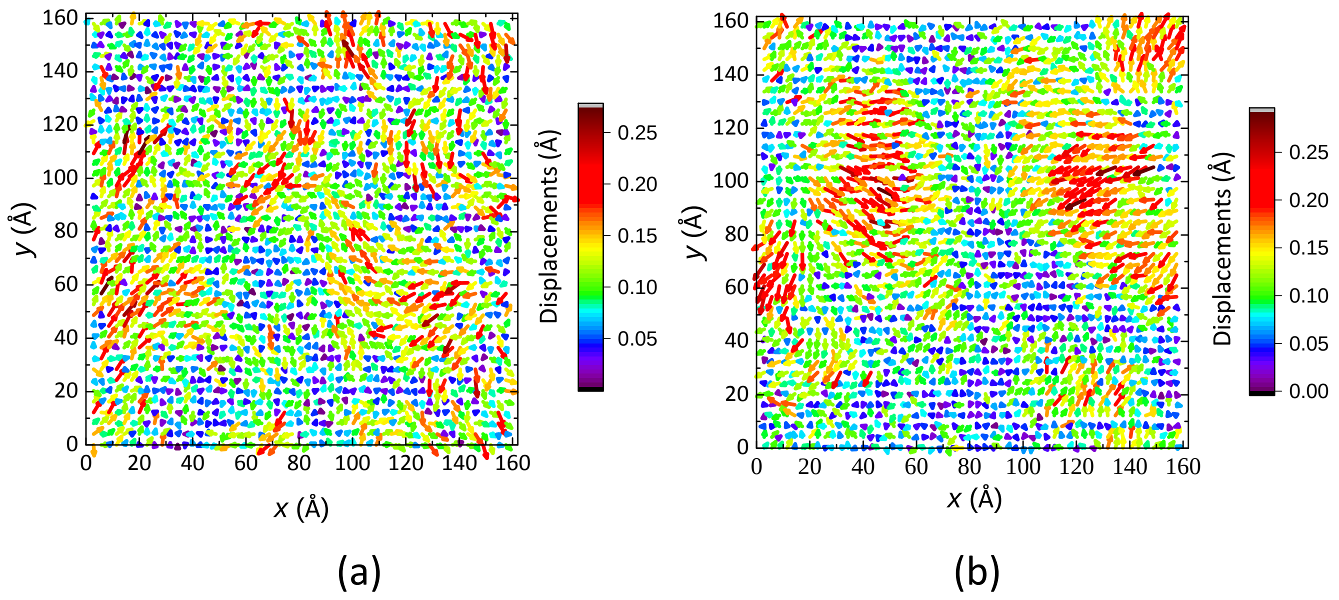

Supplementary Figure 9: Variable-temperature maps of the Pb-column displacements. Maps of displacements for the Pb columns projected onto a {110} plane for 300 K (a) and 200 K (b). Growth of PNRs at 200 K is evident. The axes *x* and *y* correspond to the orthogonal ⟨110⟩ and ⟨001⟩ directions, respectively.

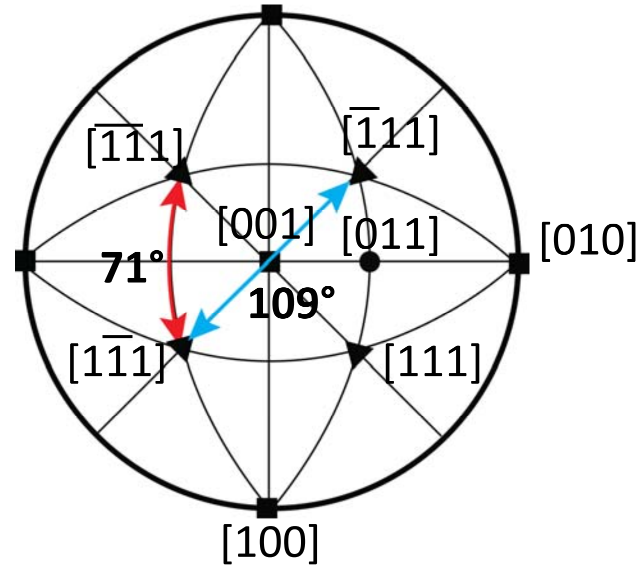

Supplementary Figure 10: A map of ⟨111⟩ variants. A stereographic-projection rendering of the 71° and 109° ⟨111⟩ variants. Vector sums for the 71°- and 109°-variant pairs are aligned with the ⟨110⟩ and ⟨100⟩ directions, respectively.

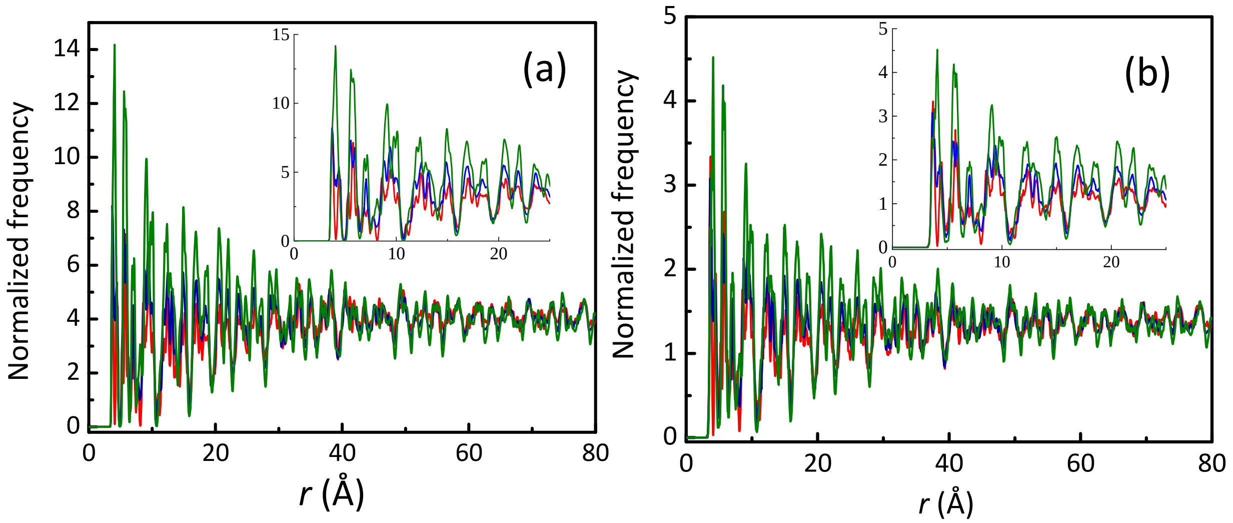

Supplementary Figure 11: Histograms of Pb-Pb distances. Histograms of distances that separate the Pb atoms displaced along the ⟨111⟩ directions (±20° tolerance) corresponding to the 71° (green), 109° (red), and 180° (blue) pairs of the ⟨111⟩ variants. A strong preference for the 71° neighboring ⟨111⟩-PNRs is evident from the much higher frequency of the distances between the Pb atoms that belong to these variants. (a) 300 K, (b) 200 K. For insets, the axes labels and scales are the same as for the main plots.

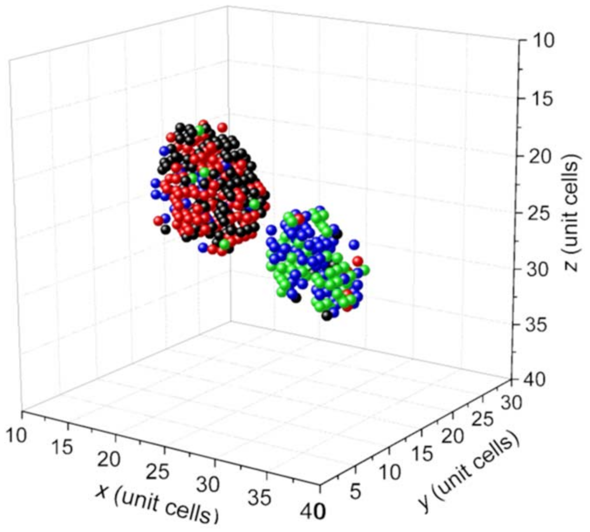

Supplementary Figure 12: Clusters yielding the characteristic diffuse scattering. 3D clusters of the Pb atoms that exhibit the strongest variation of the amplitude in the inverse FT performed using the diffuse-scattering features near the Bragg peaks. Distinct colors are used to distinguish Pb atoms that are displaced within the cones (35 semi-angle) centered on the [-1-11] (red), [-111] (black), [1-1-1] (green), and [1-11] (blue) directions, respectively. Each cluster is dominated by a pair of the 71° variants.

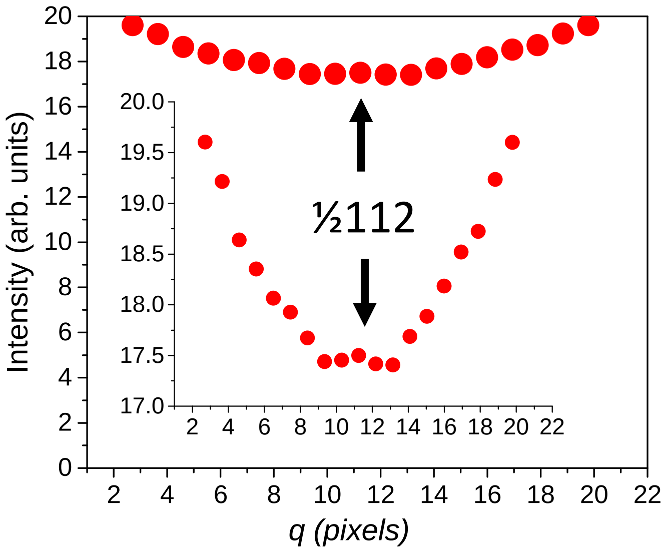

Supplementary Figure 13: A diffuse-intensity profile across the ½½1 location. An experimental diffuse-intensity profile along the line passing through the 101 and 011 reflections in the {*hk*1} section of the reciprocal space. The main plot shows the intensity on the full scale, whereas the inset displays a magnified view demonstrating the presence of the distinct ½½1 peak. For the inset, the axes labels and scales are the same as for the main plot.

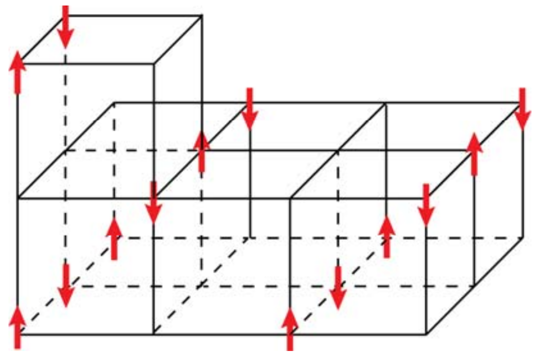

Supplementary Figure 14: Antipolar ordering of the Pb displacements. Schematic depiction of the local ordering for the anti-parallel Pb displacements as recovered using the inverse Fourier filtering of the calculated amplitude of the M-points. The Pb atoms are located at the nodes of the grid. The arrows indicate the directions of the Pb displacements.

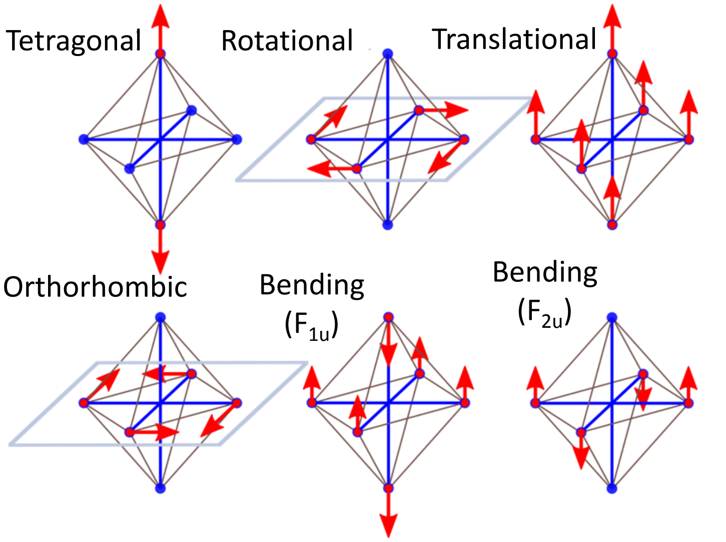

Supplementary Figure 15: Octahedral deformation modes. Schematic rendering of the octahedral deformation modes used as the basis for expanding the oxygen displacements.

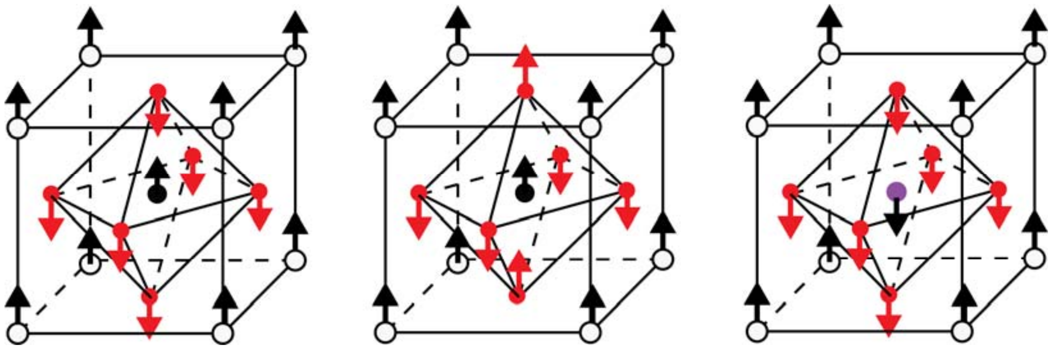

(a) (b) (c)

Supplementary Figure 16: Cation-oxygen displacement modes. Schematic rendering of the cation-oxygen displacement modes identified in the refined configuration. These modes match the typical lowest-frequency optical modes in ferroelectric perovskites. (a-b) – [NbO_6] octahedra, (c) – [MgO_6] octahedra. The open circles refer to the Pb atoms. The filled black (a, b) and purple (c) circles refer to the Nb and Mg atoms, respectively. The filled red circles forming an octahedron represent the O atoms. The arrows indicate the directions of atomic displacements.

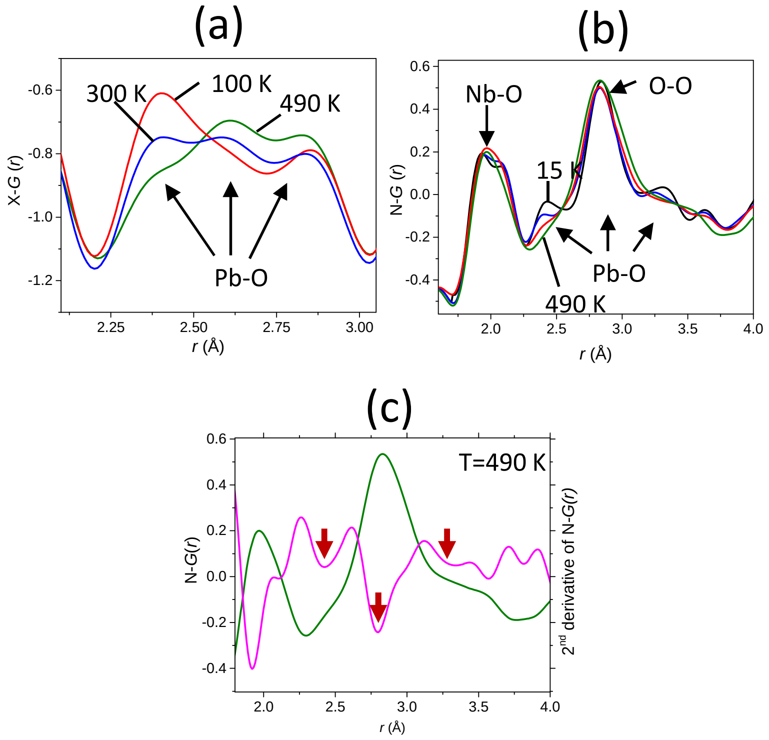

Supplementary Figure 17: (a) Pb-O peak in the X-ray PDF (X-*G*(*r*)) measured at 100 K (red), 300 K (blue), and 490 K (green). The peak splitting and the presence of short (≈2.4 Å) Pb-O distances at 490 K is evident. (b) A low-*r* portion of the neutron PDF (N-*G*(*r*) at 15 K (black), 200 K (blue), 300 K (red), and 490 K (green). The Pb-O peak splitting which yields the ≈2.4 Å distances is still present at 490 K. (c) A magnified view of the Pb-O peak in the neutron PDF at 490 K (green). The magenta trace represents the 2nd derivative of this signal which confirms the presence of the Pb-O peaks at ≈2.4 Å and ≈3.3 Å, as can be inferred from the corresponding minima (indicated using red arrows) of the derivative.

Supplementary Table 1: Deformation modes for the oxygen octahedra. Columns describing the transformations of oxygen coordinates according to the 18 orthonormal deformation modes for an oxygen octahedron. The modes are denoted as following (see Supplementary Figure 15): Tetr – tetragonal, Ort – orthorhombic, Rot – rotational, Trans – translational, Buu – bending F_{1u}, Bud – bending, F_{2u}. For each column, the sum of squared entries is equal to unity.

	TetrZ	TetrY	TetrX	OrtRZ	OrtRY	OrtRX	RotZ	RotY	RotX	TransZ	TransY	TransX	BuuZ	BuuY	BuuX	BudZ	BudY	BudX
1 x	0	0	0.70711	0	0	0	0	0	0	0	0	0.40825	0	0	-0.57735	0	0	0
1 y	0	0	0	0.5	0	0	0.5	0	0	0	0.40825	0	0	0.28868	0	0	-0.5	0
1 z	0	0	0	0	0.5	0	0	-0.5	0	0.40825	0	0	0.28868	0	0	0.5	0	0
2 x	0	0	0	0.5	0	0	-0.5	0	0	0	0	0.40825	0	0	0.28868	0	0	0.5
2 y	0	0.70711	0	0	0	0	0	0	0	0	0.40825	0	0	-0.57735	0	0	0	0
2 z	0	0	0	0	0	0.5	0	0	0.5	0.40825	0	0	0.28868	0	0	-0.5	0	0
3 x	0	0	0	0	0.5	0	0	0.5	0	0	0	0.40825	0	0	0.28868	0	0	-0.5
3 y	0	0	0	0	0	0.5	0	0	-0.5	0	0.40825	0	0	0.28868	0	0	0.5	0
3 z	0.70711	0	0	0	0	0	0	0	0	0.40825	0	0	-0.57735	0	0	0	0	0
4 x	0	0	-0.70711	0	0	0	0	0	0	0	0	0.40825	0	0	-0.57735	0	0	0
4 y	0	0	0	-0.5	0	0	-0.5	0	0	0	0.40825	0	0	0.28868	0	0	-0.5	0
4 z	0	0	0	0	-0.5	0	0	0.5	0	0.40825	0	0	0.28868	0	0	0.5	0	0
5 x	0	0	0	-0.5	0	0	0.5	0	0	0	0	0.40825	0	0	0.28868	0	0	0.5
5 y	0	-0.70711	0	0	0	0	0	0	0	0	0.40825	0	0	-0.57735	0	0	0	0
5 z	0	0	0	0	0	-0.5	0	0	-0.5	0.40825	0	0	0.28868	0	0	-0.5	0	0
6 x	0	0	0	0	-0.5	0	0	-0.5	0	0	0	0.40825	0	0	0.28868	0	0	-0.5
6 y	0	0	0	0	0	-0.5	0	0	0.5	0	0.40825	0	0	0.28868	0	0	0.5	0
6 z	-0.70711	0	0	0	0	0	0	0	0	0.40825	0	0	-0.57735	0	0	0	0	0
